# Whole-genome surveillance identifies markers of *Plasmodium falciparum* drug resistance and novel genomic regions under selection in Mozambique

**DOI:** 10.1128/mbio.01768-23

**Published:** 2023-09-26

**Authors:** Erin Coonahan, Hunter Gage, Daisy Chen, Emilia Virginia Noormahomed, Titos Paulo Buene, Irina Mendes de Sousa, Kevan Akrami, Lucia Chambal, Robert T. Schooley, Elizabeth A. Winzeler, Annie N. Cowell

**Affiliations:** 1 School of Medicine, University of California San Diego, La Jolla, California, USA; 2 Department of Pediatrics, University of California San Diego (UCSD), La Jolla, California, USA; 3 Department of Microbiology, Parasitology Laboratory, Faculty of Medicine, Eduardo Mondlane University, Maputo, Mozambique; 4 Mozambique Institute of Health Education and Research (MIHER), Maputo, Mozambique; 5 Biological Sciences Department, Faculty of Sciences, Eduardo Mondlane University, Maputo, Mozambique; 6 Faculdade de Medicina da Bahia, Universidade Federal da Bahia, Salvador, Brazil; 7 Department of Internal Medicine, Faculty of Medicine, Eduardo Mondlane University, Maputo, Mozambique; 8 Maputo Central Hospital, Maputo, Mozambique; NIAID/NIH, Rockville, Maryland, USA

**Keywords:** *Plasmodium falciparum*, severe malaria, whole-genome sequencing, selective whole genome amplification, antimalarial drug resistance, genetic variation, positive selection

## Abstract

The emergence of *Plasmodium falciparum* drug resistance threatens the efficacy of antimalarial therapies globally. Population genomic studies have been instrumental in mapping malaria genes associated with drug resistance. However, to understand the impact of regional malaria control strategies on the malaria genome, it is necessary to examine parasite subpopulations at the country level. Here, we use selective whole-genome amplification (SWGA) to analyze 120 samples from patients with severe malaria in Mozambique, a country with high malaria burden and risk of emerging drug resistance. We demonstrate that SWGA allows for the generation of near-complete whole-genome sequences from clinical *P. falciparum* isolates. Our analysis shows that Mozambique parasite genomes show strong diversification of vaccine targets including the circumsporozoite protein (*csp*), the target of the RTS,S vaccine. Parasites in this population appear fully sensitive to chloroquine, bearing no likely resistance conferring mutations in the chloroquine resistance transporter, *pfcrt* (PF3D7_0709000) and highly resistant to sulfadoxine-pyrimethamine, exhibiting resistant haplotypes at near fixation. Despite a long history of artemisinin use in Mozambique, we find no evidence of selection near *pfkelch13* (PF3D7_1343700) nor evidence of artemisinin resistance conferring mutations in *pfkelch13*. Using identity by descent analysis, we show positive selection at genomic regions on chromosomes 6, 8, 12, 13, and 14. Gene candidates within these regions include the amino acid transporter 1 (*pfaat1*, PF3D7_0629500) on chromosome 6 which bears an S258L substitution in >80% of parasites, and a region on chromosome 14 previously amplified under artemisinin pressure.

**IMPORTANCE:**

Malaria is a devastating disease caused by *Plasmodium* parasites. The evolution of parasite drug resistance continues to hamper progress toward malaria elimination, and despite extensive efforts to control malaria, it remains a leading cause of death in Mozambique and other countries in the region. The development of successful vaccines and identification of molecular markers to track drug efficacy are essential for managing the disease burden. We present an analysis of the parasite genome in Mozambique, a country with one of the highest malaria burdens globally and limited available genomic data, revealing current selection pressure. We contribute additional evidence to limited prior studies supporting the effectiveness of SWGA in producing reliable genomic data from complex clinical samples. Our results provide the identity of genomic loci that may be associated with current antimalarial drug use, including artemisinin and lumefantrine, and reveal selection pressure predicted to compromise the efficacy of current vaccine candidates.

## INTRODUCTION

Mozambique is a country of approximately 31 million people in Southeastern Africa which has the fourth highest number of malaria cases in the world (1). In 2021, malaria was the second leading cause of death in Mozambique, behind HIV/AIDS, with over 10 million malaria infections and 22,300 malaria-related deaths (2). Rates of malaria and HIV coinfection are high (up to 53% in hospitalized patients in Maputo, the country’s capital [3]) which increases patients’ risk of developing severe malaria and harboring drug-resistant parasites due to antiviral and prophylactic drug exposure (4 – 6). Despite these high number of cases, Mozambique is poorly represented in whole-genome sequencing (WGS) studies of *Plasmodium falciparum* parasites, with only one sample in the Pf6 data set (7).

In Mozambique, most malaria cases are caused by *P. falciparum*, and current first-line treatment is the artemisinin combination therapy (ACT) artemether-lumefantrine (AL) for uncomplicated malaria or artesunate (AS) for severe disease. Over the last decade, there have been reports of increasing resistance to current first-line ACTs in Southeast Asia (8 – 11), and artemisinin resistance conferring mutations in *pfkelch13* have recently been reported in Rwanda and Uganda (12 – 14). ACT treatment efficacy is still high on the African continent presumably due to ongoing efficacy of ACT partner drugs. However, the R561H mutation in *pfkelch13* has recently been associated with delayed parasite clearance following treatment with AL in Rwanda (15, 16). Increased genomic surveillance is crucial to predict future drug failures and reveal evolving mechanisms of artemisinin and lumefantrine (LF) resistance.

Mozambique follows WHO’s recommendation on surveillance of molecular markers of resistance, but few reports have been published. Previous studies on parasites in the country have reported an increase in the chloroquine (CQ)-sensitive K76 allele in the chloroquine resistance transporter (*pfcrt,* PF3D7_0709000) and an increase in the multidrug resistance protein *pfmdr1* (PF3D7_0523000) N86/Y184F/D1246 haplotype attributed to widespread AL use in recent decades (17 – 19). A recent study also reported high rate of mutations in *pfdhfr* (PF3D7_0417200) and *dhps* (PF3D7_0810800) associated with sulfadoxine-pyrimethamine resistance (19). No *pfkelch13* mutations associated with artemisinin resistance have been reported (19, 20).

Methods for assessing antimalarial drug resistance are technically challenging and time-consuming. Human trials for drug efficacy are logistically difficult, and phenotyping parasites requires sophisticated equipment and is often impractical in areas where the disease burden is highest. Whole-genome sequencing enables the detection of known resistance markers and genomic analysis to predict emerging mechanisms of resistance. However, obtaining high-quality DNA from field samples is difficult, as parasite DNA is a small fraction of DNA from human whole blood samples. Selective whole-genome amplification (SWGA) is a technique that uses specific primers to amplify parasite DNA, enriching the genome of interest prior to sequencing (21 – 24). Here, we use SWGA to analyze unprocessed clinical samples and increase genomic coverage. The protocol consists primarily of PCR, an affordable and accessible technique, which then allows for the generation of high-quality sequencing data. SWGA is a valuable tool to increase drug resistance surveillance in the African continent and enables in-country processing and data collection from complex field samples.

We obtained *P. falciparum* sequences from whole blood samples collected from patients admitted to Maputo Central Hospital in Mozambique with severe malaria infection. Through SWGA, we obtained robust whole-genome sequences that enabled identification of drug resistance markers and genomic regions in disequilibrium. First, we screened for markers of resistance in known drug resistance genes. We then assessed evidence of selective pressure on regions of the genome via identity by descent (IBD) analysis, Tajima’s *D* scoring to reveal balancing selection, and compared relative rates of missense vs synonymous mutations across genes.

## RESULTS

### SWGA allows for the cost-effective generation of reliable complete genome data from complex samples

We examined parasite DNA from human whole blood samples without leukocyte depletion. Patients had an average parasitemia of 1.1% (0.2%–15.6%), and 40.9% were coinfected with HIV. An initial subset of samples (*n* = 7) was sequenced without SWGA to assess sequence quality. The unamplified samples had an average mean coverage of 1.98× and 14.7% of bases covered by ≥5 reads, compared to an average coverage of 46.1× and 76.0% of bases covered by ≥5 reads after SWGA (Table S1). Thus, SWGA was performed on all samples (*n* = 120) to achieve adequate coverage for analysis. Coverage statistics are reported in Table 1. Read coverage generally correlated with parasitemia (Spearman’s rank correlation coefficient = 0.653, Fig. S1). After SWGA, high coverage (defined as ≥75% of the genome covered by ≥5 reads) was attained for all samples with parasitemia above 0.4% and for some samples with parasitemia as low as 0.2% (limit of parasite detection).

**TABLE 1:** Summary of sequencing results^*a*^

Average total reads per sample	35055111.3 (5,661,278–145,431,302)
Average % of reads aligned	53.7% (1.4%–98.44%)
Average mean coverage	53.5x (0.25×–168.13×)
Average genome callable (% covered by ≥5 reads)	70.8% (1.4%–98.4%)
Average genome callable, for samples with parasitemia >0.2% (% covered by ≥5 reads)	88.9% (3.2%–98.4%)
Average genome callable, for samples with parasitemia at LOD, 0.2% (% covered by ≥5 reads)	50.1% (1.4%–96.6%)

^*a*^ Sequences were obtained from 120 patients. Greater than 70% of the genome was covered by ≥5 reads across samples. Coverage was correlated with parasitemia (Fig. S1), with only 50% of the genome covered by ≥5 reads for samples with a parasitemia at the limit of detection (LOD) (0.2% or 1 in 500 infected RBCs).

**Fig 1:**
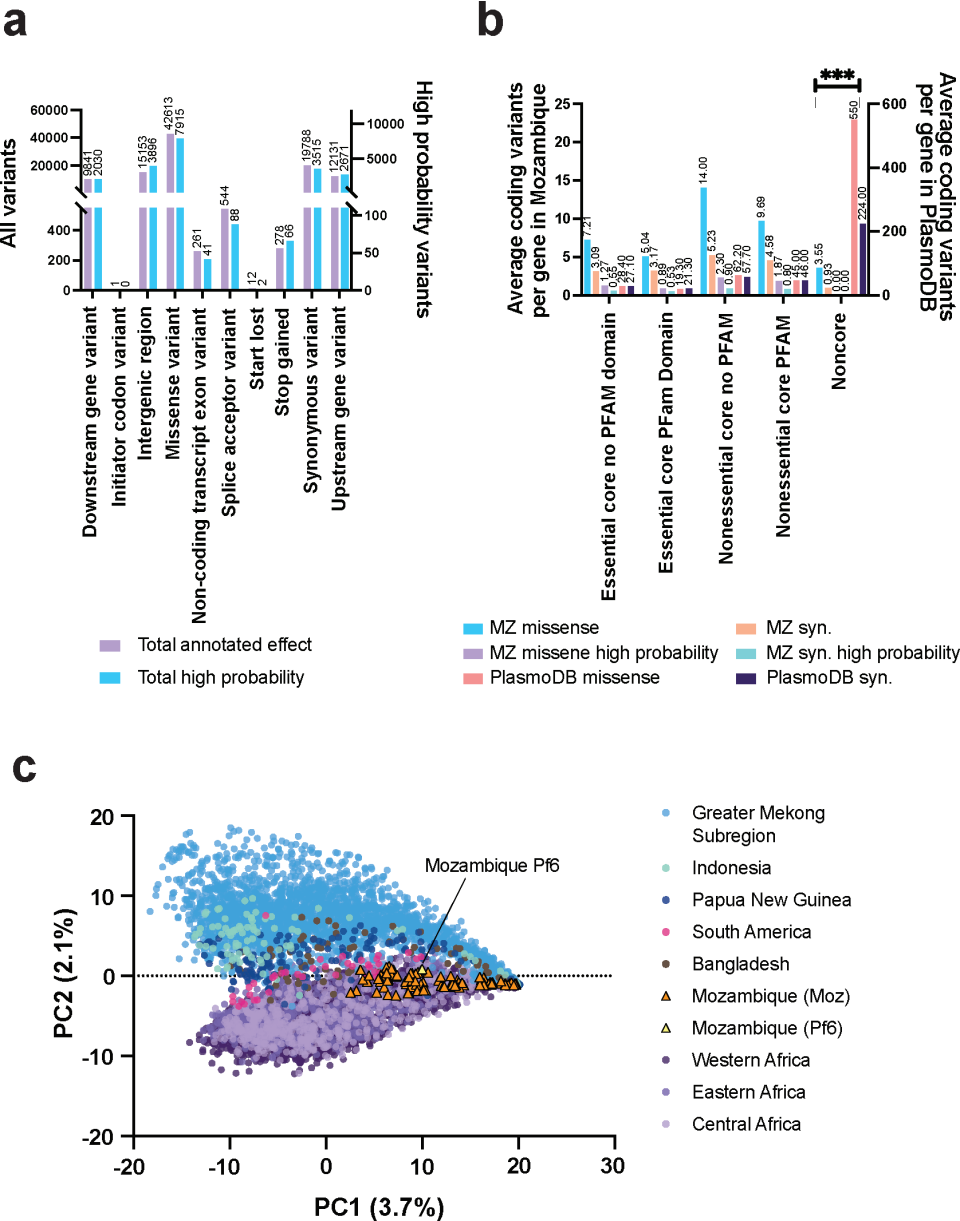
Validation of Mozambique data set. (a) Types of genic and intergenic variants detected as a homozygous call in at least one Mozambique sample (136,720), or detected at least five times (high probability, 27,070 variants) in one of the 120 samples. (b) Proportions of missense and synonymous (syn.) mutations in all (61,392) and high probability coding variants (10,567) in Mozambique samples and in variants identified in PlasmoDB release 61 (333,648 and 239,283, respectively) for 5,361 genes (mitochondrial, RNA, apicoplast, and pseudogenes removed). Genes were classified based on essentiality (25) as well as the presence of recognizable PFAM domains and were normalized based on total genes in the category. The data show a higher proportion of mutations in nonessential genes with no homology to other genes, typically classified as “conserved, unknown function” and a lower proportion in genes such as enzymes. Noncore genes were defined by Otto et al. (26) and consist largely of *var* genes, *rifin*s and *stevor*s (281 genes). (c) Principal component analysis (PCA) of Mozambique variants in relationship to variants from the Pf6 data set, comprising genome sequences for 7,113 *P*. *falciparum* samples from 28 malaria-endemic countries spanning 2001–2015 (7). PCA was performed on binary encoding of alternate allele presence in 459,725 core genome sites that were called as potential missense variants in Pf6 and were classified as present in at least one sample from the combined Mozambique and Pf6 data (see Materials and Methods).

We identified 172,627 high-quality single-nucleotide variants (SNVs) in our sample set compared to the reference genome. Filtering for SNVs homozygous in at least one sample reduced the variants to 136,720. Among these, we classified 27,070 single-nucleotide polymorphisms (SNPs) as high probability SNPs, found in at least five samples. These SNPs were further categorized as genic and intergenic. Notably, we observed a higher number of missense mutations (7,920) compared to synonymous mutations (3,593) (Fig. 1a).

Comparing missense and synonymous variants in our data set to those cataloged in PlasmoDB showed similar ratios for most gene classes (Fig. 1b) building greater certainty in our findings. We did observe fewer mutations in noncore, subtelomeric genes in Mozambique samples than expected. This could be due to spurious calls in noncore regions in PlasmoDB or lower coverage in our samples in the noncore region as these can be difficult to align and may reflect fewer SWGA primers mapping to multigene hypervariable family members such as *rifin*, *stevor,* and *var* genes. Our mean depth of coverage was calculated to be sixfold higher across the core regions of the *P. falciparum* genome compared to noncore regions, as defined by Otto et al. (26).

To further assess sequence quality, we performed principal component analysis (PCA) to visually compare our sequences to those from other regions in Africa and the world in the Pf6 data set (Fig. 1c) (7). Using binary encodings of 459,725 sites in the core genome with missense variants in Pf6 as PCA input, we found that Mozambique samples consistently cluster with the single Pf6 Mozambique sample as well as other African samples across the top eight principal components (PCs) (Fig. S2b through e); meanwhile, consistent with previous studies, Southeast Asia forms its own cluster that strongly separates from Africa in one of the top two PCs. Analysis of approximate genetic distance between samples showed that our Mozambique isolates were most similar to neighboring countries in Eastern Africa such as Malawi and Uganda (Fig. S2a). These results provide further evidence that the Mozambique sequencing results likely reflect true genomic variants and that SWGA generates reliable data for population genetic analysis.

To investigate polyclonality, the Fws statistic was calculated across core genes for 87 high coverage samples (≥75% of genome covered by ≥5 reads) using the moimix package available in R. The average Fws metric across samples was 0.87 and using an Fws cutoff of 0.95 and a cutoff for mixed calls greater than 2× the median across samples (27), we estimate that approximately 45% of samples are multiclonal (28 – 30) (Table S2).

### Known drug resistance alleles present in Mozambique

We next sought to identify mutations in known drug resistance genes. In Mozambique, sulfadoxine-pyrimethamine (SP) plus amodiaquine (AQ) was introduced in 2004, replacing CQ as the first-line treatment for uncomplicated malaria. In 2006, AS plus SP became the first-line therapy but was replaced by AL in 2008 due to increased evidence of SP resistance (31, 32). However, SP is currently used for intermittent preventative therapy (IPT) during pregnancy and is being piloted for seasonal prophylaxis in children under 5 (1). SP targets two multifunctional enzymes involved in folate biosynthesis, *pfdhfr* via pyrimethamine, and *dhps* via sulfadoxine. Mutations in the active site block drug access to confer resistance (33, 34). All samples genotyped at *pfdhfr* contain the triple mutant (N51I, C59R, S108N) (35) found in 100% of the callable sites (2). This *pfdhfr* “triple mutant” genotype is known to confer pyrimethamine resistance and have been highly prevalent in East Africa for the last 20 years (32, 35 – 37). In *dhps,* we found the resistance-conferring K540E mutation in 88 samples (of 97 callable). Of samples that were wild type at position 540, three carried resistance conferring S436C or S436A mutations (Table 2). Thus, no samples were predicted to be fully sensitive to SP, and only three are predicted to be sensitive to sulfadoxine but not to pyrimethamine.

**TABLE 2:** List of missense variants in important drug resistance genes and targets^*a*^

Gene name	Gene ID	Description	Genomic location	AA substitution (#variants/total callable)
DHFR-TS	PF3D7_0417200	Bifunctional dihydrofolate reductase-thymidylate synthase	Pf3D7_04_v3: 748,088..749,914(+)	p.N51I (95/95) p.C59R (94/96) p.S108N (93/93)
MDR1	PF3D7_0523000	Multidrug resistance protein 1	Pf3D7_05_v3: 957,890..962,149(+)	p.Y184F (39/89) p.D642G (6/86) p.N652D (39/84)
AAT1	PF3D7_0629500	Amino acid transporter AAT1	Pf3D7_06_v3: 1,213,948..1,216,005(−)	p.S258L (80/96)
PPPK-DHPS	PF3D7_0810800	Hydroxymethyldihydropterin pyrophosphokinase-dihydropteroate synthase	Pf3D7_08_v3: 548,200..550,616(+)	p.G437A (6/99) p.K540E (88/97) p.S436A/C(3/99)^*b*^
ACT	PF3D7_1036800	“Acetyl-CoA transporter 1, putative”	Pf3D7_10_v3: 1,448,692..1,451,646(−)	p.T459I (76/91) p.I140V (8/94)
MRP2	PF3D7_1229100	Multidrug resistance-associated protein 2	Pf3D7_12_v3: 1,192,888..1,199,214(−)	p.L1531V (69,2/94) p.S1527T (74/94) p.S1371N (9/97) p.K714I (81/98) p.L199V (98/98)
CRN	PF3D7_1251200	Coronin	Pf3D7_12_v3: 2,092,087..2,094,231(+)	p.S183G (73/94) p.V424I (6/99) p.F434L (5/99)
K13	PF3D7_1343700	Kelch protein K13	Pf3D7_13_v3: 1,724,817..1,726,997(−)	p.K189T (10/91)
MDR2	PF3D7_1447900	Multidrug resistance protein 2	Pf3D7_14_v3: 1,954,601..1,957,675(−)	p.S635T (7/98) p.I492V (13/97) p.F423Y (19/99) p.S208N(79/93)

^*a*^ Numbers of alternate alleles are shown in parentheses with total callable sites (of 120). Only alleles with an alternate reference found in at least five samples are shown with the exception of *pppk-dhps*.

^*b*^ Sensitive genotype: reference strain 3d7 is sulfadoxine resistant with an A437G mutation (33).

Mutations in *pfmdr1* confer resistance to multiple antimalarial treatments. The N86Y mutation is associated with resistance to CQ and AQ but sensitivity to mefloquine (MQ) and LF. We did not find N86Y mutations in our Mozambique cohort. Parasites with the wild-type N86 and *pfmdr1* haplotype N86/Y184F/D1246 are selected for by widespread use of AL (18, 38, 39). The Y184F mutation detected in 39 of our samples is associated with CQ and AQ resistance (40), often in concert with N86Y and mutations in *pfcrt* (38, 41). We also found two new mutations in *pfmdr1*, N652D in 39 samples and D642G in 6 samples (Table 2). These mutations have not been linked to multidrug resistance, and both are located in poorly conserved repetitive domains away from the critical channel region.

Mutations in *pfcrt* are known to confer resistance to chloroquine. The *pfcrt* K76T mutation is the most well known and confers resistance to AQ as well (42). *Pfcrt* alleles were common in African isolates 20 years ago, but many parasites in the region have reverted to wild type in recent years in the absence of quinoline drug pressure and widespread AL use which selects for the wild-type K76 allele (43, 44). Examining *pfcrt,* we found only a single, low probability missense D24Y mutation in one sample, located away from the channel known to confer drug resistance and no K76T mutations. To further confirm these negative results in *pfcrt*, we examined read coverage over the gene (chr7:403K-407K) across all samples and found a sufficient mean depth of coverage of 4127.28× (Table S3).

Specific mutations in *pfkelch13* are known to cause an artemisinin-delayed clearance phenotype (45). Most characterized resistance conferring mutations, including the well-characterized C580Y mutation, are in the propeller domain from amino acid 442 to 727 (45, 46). In Africa, the A675V and C469Y *pfkelch13* mutations have been associated with delayed clearance after artemisinin treatment (11). We identified one high probability *pfkelch13* mutation in 10 samples (Table 2), but this was located at position 189 and thus is less likely to be associated with resistance. We also found no evidence of mutations in the propeller region of *coronin* (PF3D7_1251200), which codes for another protein that can be mutated to confer artemisinin tolerance (47).

### Identity by descent mapping identifies regions under selection

To identify novel loci that are under selection in Mozambique, we scanned the genome for regions with a high degree of IBD. Two alleles are considered identical by descent if they were inherited from a common ancestor. Closely related individuals tend to have long, frequently distributed regions of IBD sharing. As recombination occurs within a population over time, the IBD segments become shorter and are less frequently distributed throughout the genome. However, selected alleles become common more quickly than recombination can reduce linkage disequilibrium and therefore have a higher degree of IBD sharing than would be expected under neutrality (48). The concept of IBD has been used in human studies to investigate disease mapping (49) and familial relatedness (50), and has more recently been used to study the population structure (51 – 53), transmission dynamics (51 – 53), and loci under selection (54, 55) in *Plasmodium*. Our analysis identified five peaks with a proportion of IBD sharing above a threshold of 0.0172 on chromosomes 6, 8, 12, 13, and 14 (Table 3). Our data showed some of the same areas that are under selection in parasites from other African regions, including peaks near *dhps*, *pfaat1*, GTP cyclohydrolase 1 (*gch1,* PF3D7_1224000), and multidrug resistance-associated protein *2* (*pfmrp2,* PF3D7_1229100) (54).

**TABLE 3:** Regions of the genome with high levels of IBD sharing^*a*^

Region of high IBD sharing	Gene ID	Description	Start	End
CHR 6 (PF3D7_6:1,094,803–1,263,547	PF3D7_0629500	Amino acid transporter 1	1,213,948	1,216,005
CHR 8 (PF3D7_8:466,571–483,850)	PF3D7_0810800	Hydroxymethyldihydropterin pyrophosphokinase-dihydropteroate synthase	548,200	550,616
CHR 12 (PF3D7_12:776,039–910,983)	PF3D7_1224000	GTP cyclohydrolase 1	974,226	976,097
	PF3D7_122910	Multidrug resistance-associated protein 2	1,192,888	1,199,214
CHR 13 (PF3D7_13:73,919–87,2914)	PF3D7_1317000	U4/U6.U5 tri-snRNP-associated protein 2, putative	708,346	710,268
	PF3D7_1317100	DNA replication licensing factor MCM4	711,924	711,943
	PF3D7_1317200	AP2 domain transcription factor AP2-FG, putative	721,961	729,979
CHR 14 (PF3D7_14:1,737,499–2,703,922)	PF3D7_1462500	Conserved *Plasmodium* protein, unknown function	2,544,824	2,549,785
	PF3D7_1462600	Conserved *Plasmodium* membrane protein, unknown function	2,550,692	2,552,432

^*a*^ Segments of the genome with a proportion of pairs IBD >0.0172 (approximately the peak of chromosome 8) are listed. Candidate genes in and around the regions of high IBD sharing are shown.

**Fig 2:**
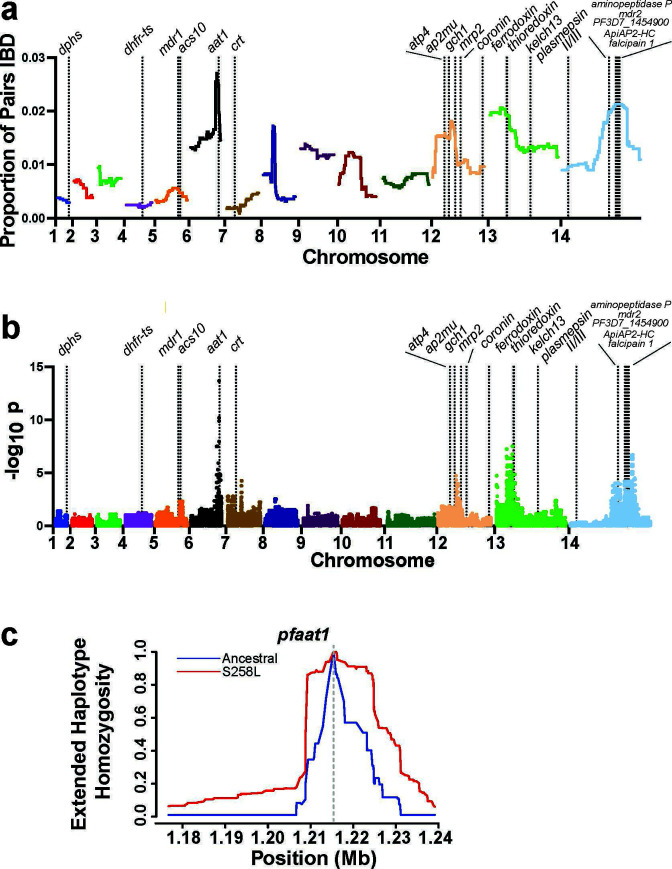
Selection signals detected via identity by descent and extended haplotype homozygosity. (a) The *isoRelate* R package was used to scan for regions of the genome with a high proportion of sample pairs that were IBD and to calculate the statistical significance of the IBD sharing. (b) The upper *x*-axes are annotated with the locations of genes previously found to be under selection. Significant IBD sharing (−log_10_ *P* > 5) was detected near *pfaat1* and regions of chromosomes 13 and 14 (candidate genes listed in Table 3). (c) The extended haplotype homozygosity in *pfaat1* decays more slowly around the S258L allele (red) relative to the ancestral allele (blue), suggesting that it is under selection.

**Fig 3:**
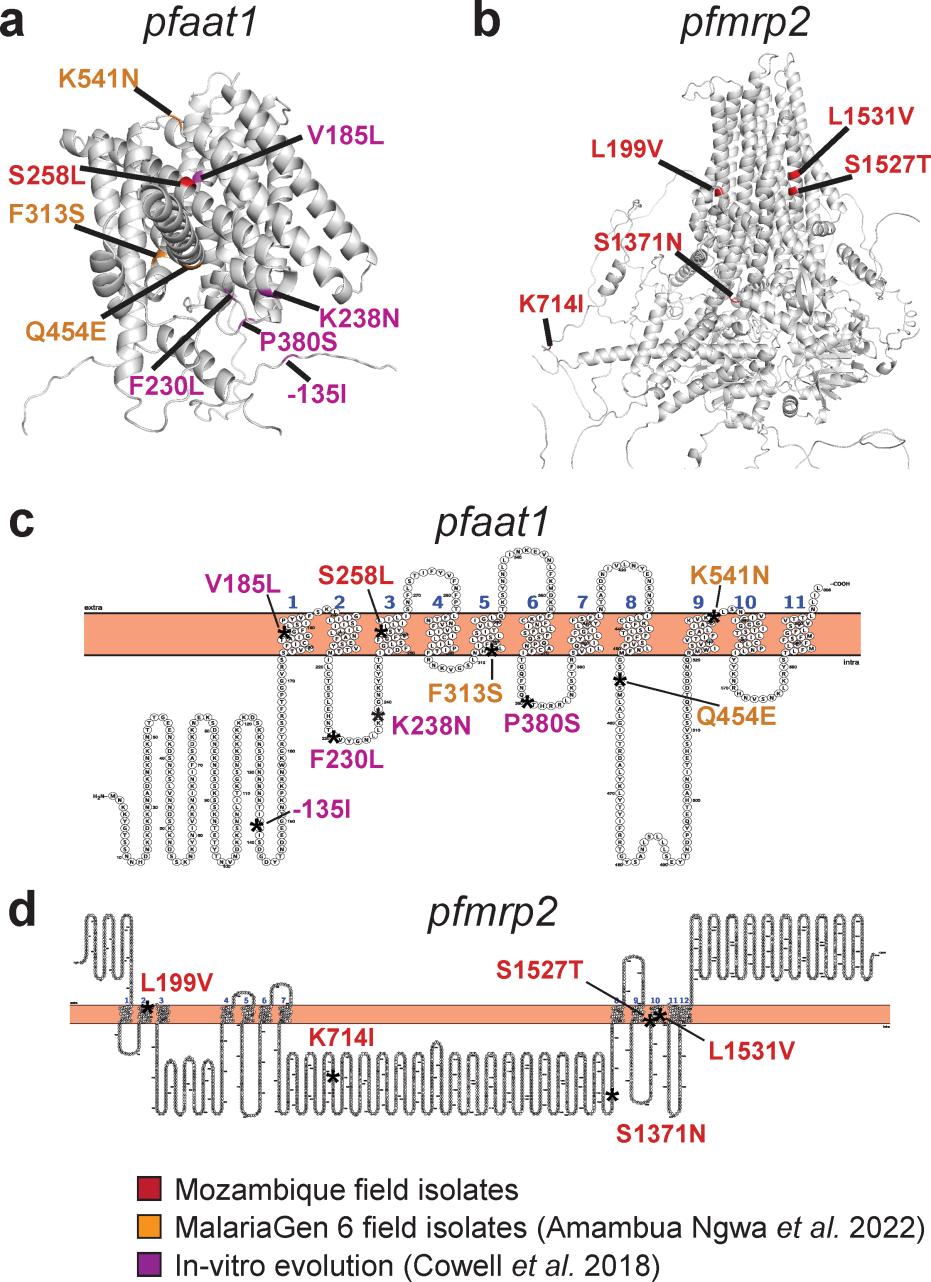
Mutations in p*faat1* and p*fmrp2* observed in field studies and *in vitro* evolution experiments. (a) AlphaFold homology model of *pfaat1*. Mutations from *in vitro* evolution experiments are highlighted in purple (59). Mutations observed in field isolates from the Pf6 database are highlighted in orange (58). Mutations observed in Mozambique are highlighted in red. The S258L mutation was present in both Mozambique and field isolates from the Pf6 database. (b) AlphaFold homology model of *pfmrp2* highlighted with mutations observed in Mozambique field isolates. (c and d) TOPCONS topology models show that many of the observed mutations are in the transmembrane channel portions of the *pfaat1* and *pfmrp2* proteins, respectively.

A likely candidate for the peak on chromosome 6 is amino acid transporter *pfaat1* (Fig. 2), which is located in the parasite food vacuole and is hypothesized to transport quinoline drug compounds and aromatic amino acids across the food vacuole, decreasing parasite exposure and sensitivity to CQ (56, 57). The *pfaat1*-S258L mutation rose in prevalence during years of CQ drug pressure in Africa in conjunction with *pfcrt*-K76T (58). *In vitro* studies suggest that this mutation, which is in the protein transmembrane domain (Fig. 3a and c), helps modulate the effect of K76T on CQ resistance, resulting in a 1.5-fold increase in the CQ IC_50_. However, this decreased drug susceptibility occurs at a significant fitness difference compared to wild-type *pfaat1 (58*). As such, it would be hypothesized that the *pfaat1* genotype would revert to majority wild-type alleles in the absence of chloroquine drug pressure. Isolates in this study carry no *pfcrt*-K76T mutations and no evidence of selection around *pfcrt*, yet they maintain *pfaat1*-258L mutations at a rate of 83.3% (80/96), suggesting that the fitness effect is dependent on the presence of the K76T allele or that alternative selection pressure exists to maintain the S258L allele. Interestingly, recent studies have reported an increased LF and AQ IC_50_ associated with the *pfaat1*-258L allele (58).

We further investigated this selection signature by calculating the extended haplotype homozygosity (EHH) around the *pfaat1*-S258L allele. EHH at a distance *x* is defined as the probability that two randomly chosen chromosomes carrying the core SNP of interest are identical by descent for the entire interval from core SNP to distance *x* (60). The EHH decays as *x* increases due to increased frequency of recombination. Regions of the genome that are under positive selection are expected to have longer haplotypes and show a delay in the decay of EHH. Indeed, we observed that the EHH decayed more slowly around the *pfaat1*-S258L allele relative to the ancestral allele (Fig. 2c), further suggesting recent selection in *pfaat1*.

The region on chromosome 12 with evidence of selection by IBD (Fig. 2) contains *pfmrp2* (encoding the multidrug resistance-associated protein 2), an ATP-binding cassette subfamily C member 2 transporter with an ortholog in humans that modulates sensitivity to antiretroviral drugs and atovaquone (61 – 63). In our data set, an L199V mutation in the channel region of *pfmrp2* expected to be associated with resistance (Fig. 3b and d) is present at fixation (98 samples). In addition, two other transmembrane variants are present, position 1531 (with two different alleles) and position 1527 (Table 2). Two 3′ UTR variants (chr 12: 1192247 and 1192242) are fixed in the population as well (94 samples). This general chromosome 12 region is also under selection in other African *P. falciparum* isolates (64) and is not far from *pfap2mu* (PF3D7_1218300) (65, 66), although this gene did not bear any missense variants in greater than two samples. The region is also near GTP cyclohydrolase, amplifications of which are associated with antifolate resistance (67). Despite the use of SWGA, we observed paired-end discordant read evidence of a 5-kb tandem duplication event involving GTP cyclohydrolase in our samples (e.g., Fig. S3).

The chromosome 13 locus has been observed in analyses by other groups, including Miotto et al., in recent samples from Papua New Guinea and Southeast Asia (68, 69). It has been proposed that a D193Y variant in *ferredoxin* (PF3D7_1318100) may confer artemisinin resistance owing to an association with artemisinin-delayed clearance phenotype (68). *Ferredoxin* did not bear any high probability missense mutations though we identify a variant downstream of the 3′ UTR present in 95 samples. It is also possible that there is a copy number variant (CNV) driving selection in the region of this peak which we could have missed as CNVs are difficult to assess after amplification with SWGA.

No obvious candidates were present on the narrow chromosome 14 IBD locus. The *plasmepsin II/III* (PF3D7_1408000) gene locus on chromosome 14 associated with piperaquine resistance (70) is located on the left arm of the chromosome, away from the selected region. *Pfmdr2* (PF3D7_1447900) is an attractive gene candidate with mutations in residues 492 and 423 located in compelling regions. *Pfmdr2* mutations are frequently found in *in vitro* evolution experiments (59) in response to drug pressure from multiple compounds. For example, *P. chabaudi* K392Q has appeared in *in vivo* evolution experiments with pyrimethamine (71), and the F423Y mutation is associated with pyrimethamine resistance in Africa (72). The I492V mutation was identified in Uganda, but its role in drug resistance is unknown (12). Genome-wide association studies show that this region is also under selection in Southeast Asia where a T484I mutation in *pfmdr2* is associated with slow clearance after artemisinin treatment (68).

It is again possible that there is a CNV in the region. *In vitro* evolution experiments with artemisinin resulted in a chromosome 14 CNV that lies directly under the broader chromosome 14 peak (73). This large artemesinin-evolved CNV stretches from position 2218830 to 2389745 and bears 20 genes, several of which have roles in hemoglobin digestion (*aminopeptidase P* [PF3D7_1454400] and *falcipain 1* [PF3D7_1458000]), free radical biology (*thioredoxin* [PF3D7_1457200]) or transcriptional responses (the *ApiAP2-HC* transcription factor [PF3D7_1456000]). Several of these genes have high-frequency missense alleles in Mozambique with one of the most intriguing proteins encoded by the small uncharacterized, highly conserved gene, PF3D7_1454900, which bears three independent missense mutations at cysteine 143. This same chromosome 14 locus has also appeared in IBD studies in other African parasites (64).

We also searched regions under selection for gene candidates that have been detected in *in vitro* evolution experiments as potential novel drug resistance or drug target candidates (59). In 15 samples, we find an M300I mutation in acyl-coA synthetase (*pfacs10*, PF3D7_0525100)*,* a promising antimalarial drug target and investigational compounds targeting this enzyme have been selected for the same mutations (74).

### Tajima’s *D* and dN/dS ratios to characterize selection

**Fig 4:**
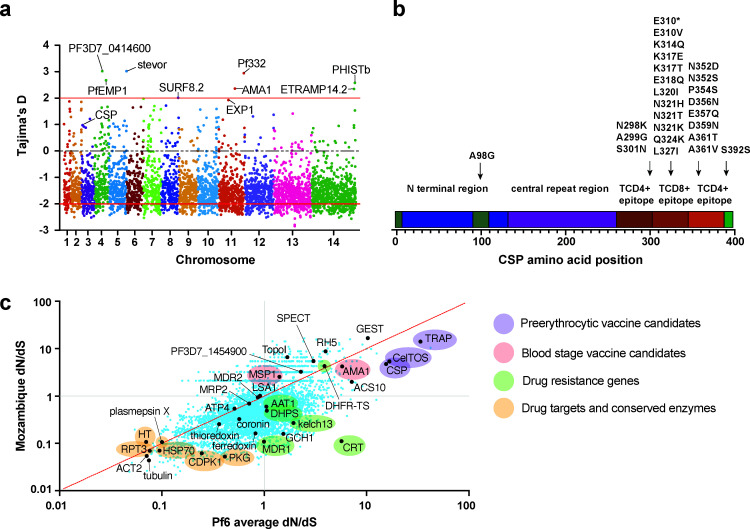
Genes under selection as determined by Tajima’s *D* and dN/dS scores. (a) Tajima’s *D* values plotted by chromosome to reveal genes under balancing selection, revealing high scores for targets of vaccines in development and testing. Drug resistance genes studied have moderately negative scores as expected since high mutation rates in single or only a few SNPs are not expected to drive large Tajima’s *D* scores. They are reported here: pfaat1 (0.102979664), pfdhfr (−1.1736371), pfdhps (−1.040123), pfcrt (−1.5699201), pfmdr1 (−0.20613), and pfkelch13 (−1.3879097). (b) Missense mutations identified in *csp* map to T-cell epitopes. (c) dN/dS (ratio of missense [nonsynonymous{NS}] to synonymous SNV counts normalized by expected numbers of NS and S sites under a neutral model) for 94 Mozambique samples with sufficient coverage vs 5,969 QC pass Pf6 samples (excluding samples from Mozambique) for all genes with at least one SNV (4,468 genes). Ratios are estimated using pseudocounts for genes with no synonymous changes.

To further characterize selection pressure, we used the Tajima’s *D* score to identify areas of the genome under balancing selection. Tajima’s D is a statistical test used to detect departures from neutral evolution in a population based on genetic variation. Genes with a positive Tajima’s D score are classified as under balancing selection and thus seeking to maintain genetic diversity. Many of the genes we report with the highest Tajima’s D scores are those targeted by the host immune system, including merozoite surface proteins, erythrocyte binding antigens, and apical membrane antigen 1 (*ama1)* (75) (Fig. 4a). Many of these are also current targets for vaccines in development, such as *ama*1 and the circumsporozoite protein (*csp),* which had Tajima’s D scores in the top 3% of all genes. *Csp* is the target of the RTS,S vaccine, and genetic variation is concerning since the vaccine is found to be less effective for *csp* alleles different from the vaccine reference genome (7, 76). In our data set, we find two samples containing mutations at amino acid position 299, reported to be associated with decreased RTS,S efficacy (76), and we find most missense mutations map to T-cell epitopes (Fig. 4b). High Tajima’s *D* scores were also reported for vaccine targets *ama1* and *exp1* (77, 78) (Fig. 4a). Many of the *P. falciparum* genes, including studied drug targets, were found to have Tajima’s *D* scores in the range of 0 to –1.5 similar to previous studies, suggesting recent population expansion or selective sweeps (79, 80) (Fig. 4a).

We next identified genes with evidence of selection based on a high proportion of missense relative to synonymous changes (dN/dS ratio), comparing our results to ratios found in the Pf6 data set (Fig. 4c). For comparison, we also analyzed dN/dS ratios of parasites from Kenya, Senegal, and Vietnam available in the pf6 database against the global pf6 data (Fig S4). These results indicated that the targets of vaccines such as *celtos*, *csp*, and *ama1* show the highest rates of missense substitution on a genome-wide scale. In fact, most vaccine candidates (81) showed high rates of selection with the exception of *msp3*. Many validated drug targets, except for targets of drugs in clinical use, such as *dhfr* and *dhps*, showed dN/dS ratios less than 1 (Fig. 4c). This is not unexpected as selection of a single missense mutation such as the S258L mutation in *pfaat1* will not drive a high dN/dS ratio. Comparing our results to Pf6 (7) samples showed similar patterns across the world with notable exceptions, including *pfcrt* and *pfmdr1,* which showed fewer missense changes in Mozambique. On the other hand, *dhfr* showed a higher dN/dS ratio than expected in both Mozambique likely reflecting the continued seasonal use of SP or drug prophylaxis in the HIV population. Topoisomerase I (Top1, PF3D7_0510500) had a rate of missense mutations in Mozambique. While not the known target of any commonly used antimalarials, *Top1* mutations could provide resistance to fluoroquinolone antibiotics such as ciprofloxacin which targets the bacterial topoisomerase, gyrase B, and is commonly taken by patients with HIV infection. Ciprofloxacin has an IC_50_ of 5.6 µM against parasites grown in culture (82), which could result in weak selection pressure.

## DISCUSSION

Mozambique has one of the highest malaria burdens in the world, and the emergence of parasite resistance to current first-line therapies could have devastating results for the country and for global progress toward malaria elimination. High transmission, challenges implementing malaria control strategies across a diverse country, and recent reports of parasite resistance on the African continent necessitate careful surveillance of the risk of emerging resistance in Mozambique.

Our data demonstrate that SWGA is a powerful technique that improves the ability to study field samples in malaria-endemic regions. SWGA increased our mean coverage by more than 20-fold, improving coverage in low parasitemia samples and increasing surveillance of an important sample population in high transmission areas. However, genome coverage was still suboptimal in some low parasitemia samples likely due to variation in sample quality and could possibly be improved with optimizing sample collection and preparation. SWGA consists primarily of PCR and can allow for in-country sample preparation and more cost-effective WGS, enabling local researchers in high malaria burden countries to collect and analyze data with less reliance on international partners. An important limitation to note is that SWGA inhibits the detection of CNVs as it inevitably amplifies some parts of the genome more than others. SWGA can also selectively amplify one clone of a multiclonal infection, skewing clonality calculations toward a higher proportion of monoclonal samples; however, previous studies have shown this effect to be minimal (23). SWGA’s ability to provide sufficient coverage for population genomic analysis is crucial for identifying emerging markers of resistance rather than simply screening known regions of interest in the genome. This is important now as parasite sensitivity is evolving on the African continent. SWGA can improve surveillance capabilities in low transmission settings and access to data among local research teams.

In our analysis of known markers of drug resistance, we found molecular evidence of high levels of SP resistance. The use of SP + AQ for chemoprevention in children and IPT in pregnancy, as well as co-trimoxazole for HIV prophylaxis (16.7% of subjects reported taking co-trimoxazole) likely contribute to the maintenance of these mutations (83). We reported no mutations in *pfkelch13* or *coronin* propeller domains, suggesting artemisinin sensitivity for the time being or perhaps a different mechanism of resistance, potentially mapping to the region under selection on chromosome 13. However, in addition to artemisinin, sensitivity to ACT partner compounds is essential to maintain efficacy of combination therapies. AL has remained effective in Mozambique without reported failures. However, with evidence of decreased artemisinin sensitivity on the continent, it is more important than ever to identify molecular markers of LF resistance to predict and intervene with alternate therapies before impending clinical failures of this first-line therapy.

To screen for novel mechanisms of resistance, we analyzed areas of the *P. falciparum* genome that are under selective pressure. Interestingly, IBD and EHH analysis showed selection around *pfaat1*, more specifically the S258L allele, which was found in>80% of our samples. *Pfaat1* has previously been implicated in modulating chloroquine sensitivity. However, the absence of CQ exposure and absence of K67T mutations suggest other survival benefits to the maintenance of this mutation, perhaps transportation of other quinoline antimalarials such as AQ or other compounds like LF (58).

Finally, our analysis shows high rates of balancing selection on genes currently targeted for vaccine development. While Mozambique was involved in early RTS,S trials, it seems more likely that this selective pressure is originating from the host immune system, unrelated to vaccine exposure. However, some polymorphisms generated by this pressure have been associated with decreased vaccine efficacy, including several mutations in *csp* found in this study. The high dN/dS ratios we observed for *csp, celtos, ama1,* and other vaccine targets further suggest selective pressure on and variability in many leading vaccine candidates with implications for vaccine success.

In summary, we use SWGA to provide an in-depth assessment of prevalence of molecular markers in Mozambique, a country with a high malarial burden and limited previous surveillance via WGS. We establish current rates of known mutations, identify areas of the parasite genome currently under selection, and examine parasites from a population that is at increased risk for harboring drug resistance mutations. We also reveal high rates of balancing selection on current vaccine targets in these samples, resulting in polymorphisms associated with decreased vaccine efficacy. Our analysis of *P. falciparum* in Mozambique provides an important update on the parasites’ ongoing attempts to evade our interventions in the fight toward malaria elimination.

## MATERIALS AND METHODS

### Sample collection

We collected 3–5 mL of whole blood from nonpregnant adult patients (>18 years) with severe malaria admitted to Maputo Central Hospital from November 2017 to April 2018. Severe malaria was defined per WHO criteria (84). The study received approval from the National Bioethics Committee for Health of Mozambique and the Human Research Protections Program of the University of California, San Diego. Patients were identified in the emergency room via positive malaria antigen or blood smear. Two independent technicians read malaria smears to determine parasite density, and a third blinded microscopist examined any discordant results.

### Generation of WGS data and variant calling

Frozen blood from 120 subjects was collected for WGS, and *P. falciparum* DNA was enriched via SWGA following previously optimized protocols (22). Sequencing libraries were prepared with Illumina’s Nextera XT Kit using the standard dual index protocol. DNA libraries were clustered and run on an Illumina HiSeq 4000 to create paired-end sequencing libraries 100 bp in length. Reads were aligned to the *P. falciparum* 3D7 reference genome following the Platypus pipeline (85), and SNPs and insertions and deletions (indels) were called using Genome Analysis Toolkit’s (GATK) HaplotypeCaller (86 – 88). The GATK Genotyper tool was used to filter variants by quality parameters, and SNPs were excluded based on the following criteria: quality depth <2.0, mapping quality <60.0, ReadPosRankSum <−8.0, phred-scaled probability of strand bias>60.0, or symmetric odds ratio >4.0. SNPs were annotated with SnpEff (v5.1 [89]).

### PCA and comparisons of variant data to the Pf6 data set

PCA was performed on binary encoding of alternate allele present in 459,725 sites in 6,686 samples, based on variant calls for this study’s Mozambique samples and preprocessed SNPs and short indels from the publicly available Pf6 data set (7). A universal strict threshold of ≥0.9 alternate allele frequency and ≥20 alternate allele depth were used for the binary encoding; as such, intermediate frequency alleles such as from polyclonal samples were not considered “confident alternate alleles” for this analysis. The 459,725 core genome sites included were called as potential missense variants in Pf6 and classified as having a confident alternate allele according to the binary encoding in at least one sample. The original 7,233 samples (120 from Mozambique, 7,113 from Pf6) were also filtered to 6,686 samples with ≥10 confident alternate alleles.

To compare Mozambique isolates with other samples in Pf6, we approximated genetic distance by taking the Hamming distance of rounded alternate allele frequencies (threshold 0.5) for 61,630 sites with minimum coverage of 20 in 4,352 samples. For country comparisons, we examined the minimum, median, and average of pairwise distances between all samples in each country.

### Modeling mutations in pfaat1 and pfmrp2 observed in field and *in vitro* studies

The AlphaFold structure predictions of the *pfaat1* and *pfmrp2* proteins (UniProt accessions C6KTD0 and Q8I5C7) (90) were visualized in PyMOL (The PyMOL Molecular Graphics System, v2.0, Schrödinger, LLC). The TOPCONS web application was used to generate a consensus prediction for transmembrane helix positions of *pfaat1* and *pfmdr2* (91). These positions were imported into the Protter web application to create 2D topology models of the transmembrane domains (92).

### Identity by descent analysis and scans for selection

To scan for regions of the genome with high levels of iIBD, the isoRelate package (54) was run in R. SNPs that were not in the *P. falciparum* 3D7 core genome (26) had missing calls in >50% of the samples or >50% missing calls across all SNPs, or had a minor allele frequency <10% were excluded. The isoRelate model has been developed and validated for detecting IBD segments in the presence of multiclonal infections and heterozygous calls. Therefore, samples with multiclonal infections were not excluded from this analysis. EHH around the pfaat1 S258L SNP was calculated using the rehh package (93) in R. SNPs that were not in the core genome or had missing calls in >25% of the samples were excluded. The EHH values were calculated for all samples by calling the calc_rehh function with default parameters (94, 95).

### Tajima’s *D* and dN/dS calculation

To assess for areas of the genome under balancing selection, we calculated the Tajima’s *D* statistic (96) for each core gene using the windowed_tajima_d function in the scikit-allel Python package (97). To estimate dN/dS, we computed numbers of missense sites (NS_exp_) and synonymous sites (S_exp_) per gene by taking the exons from protein-coding genes in the Pf3D7 genome, determining the expected number of missense (counting nonsense) vs synonymous substitutions per site. To obtain real valued dN/dS estimates for genes with zero synonymous SNVs, we defined gene-specific pseudocount equal to 1/[(S_exp_/2) + NS_exp_]. dN/dS was then calculated as the number of missense SNVs per missense site divided by the number of synonymous SNVs per synonymous site, with pseudocount added to both dN and dS. SNVs with minimum alternate allele depth of 50 and alternate allele frequency of 0.5 were included.

## Data Availability

The data sets supporting the conclusions of this article are included within the article, and its supplemental files and full sequencing data from all 120 samples are available on the National Center for Biotechnology Information’s Short Read Archive with the accession number PRJNA953166.

## AFTER EPUB

*[This article was published on 26 September 2023 with Fig. 2 inadvertently duplicated in place of Fig. 3. Figure 3 was corrected in the current version, posted on 5 October 2023.]*

## SUPPLEMENTAL MATERIAL

Fig. S1 mbio.01768-23-s0001.tif Mean coverage versus parasitemia.

Fig. S2 mbio.01768-23-s0002.tif Comparison to Pf6 samples using approximation of genetic distance and PCA.

Fig. S3 mbio.01768-23-s0003.tif Paired-end discordant read evidence of a 5kb tandem duplication event involving GTP cyclohydrolase on chromosome 12.

Fig. S4 mbio.01768-23-s0004.tif dN/dS comparisons between specific countries (Mozambique, Senegal, Kenya, and Vietnam) and worldwide averages from Pf6.

Supplemental material mbio.01768-23-s0005.docx Supplemental legends, Table S1, and Table S3.

Table S2 mbio.01768-23-s0006.xlsx Clonality calculations.

ASM does not own the copyrights to Supplemental Material that may be linked to, or accessed through, an article. The authors have granted ASM a non-exclusive, world-wide license to publish the Supplemental Material files. Please contact the corresponding author directly for reuse.
