# Supplementary figures and images for: Whole-genome surveillance identifies markers of Plasmodium falciparum drug resistance and novel genomic regions under selection in Mozambique
Source: mBio. 2023 Sep 26;14(5):e01768-23. doi: 10.1128/mbio.01768-23 (PMC10653802; doi:10.1128/mbio.01768-23)

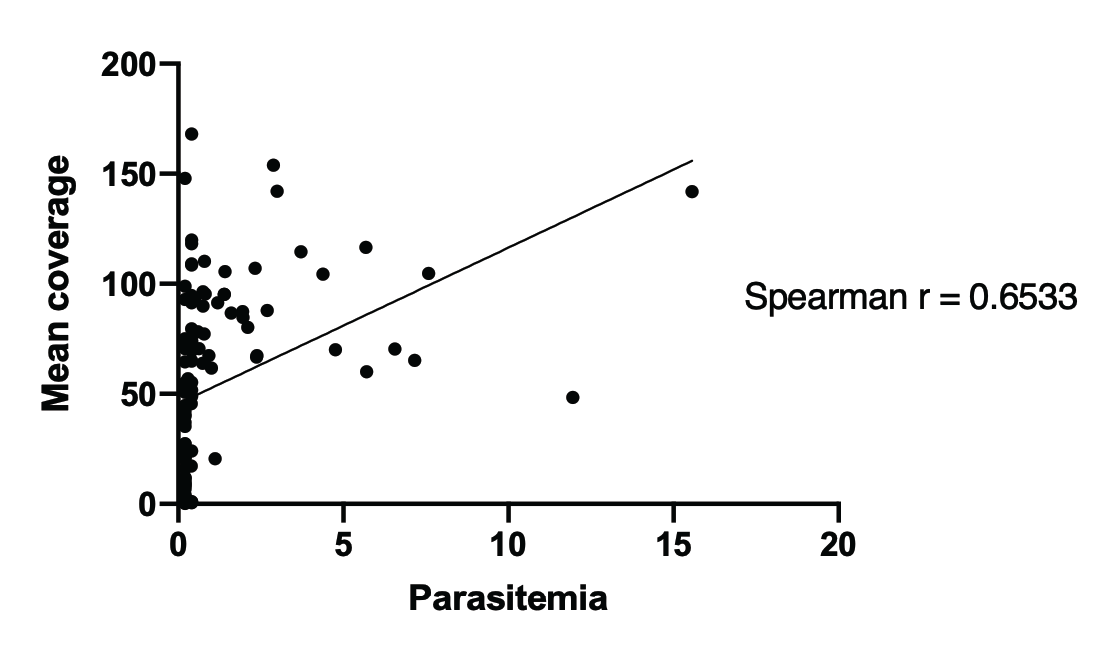

Supplement: Fig. S1 — Mean coverage versus parasitemia. [file mbio.01768-23-s0001.tif]

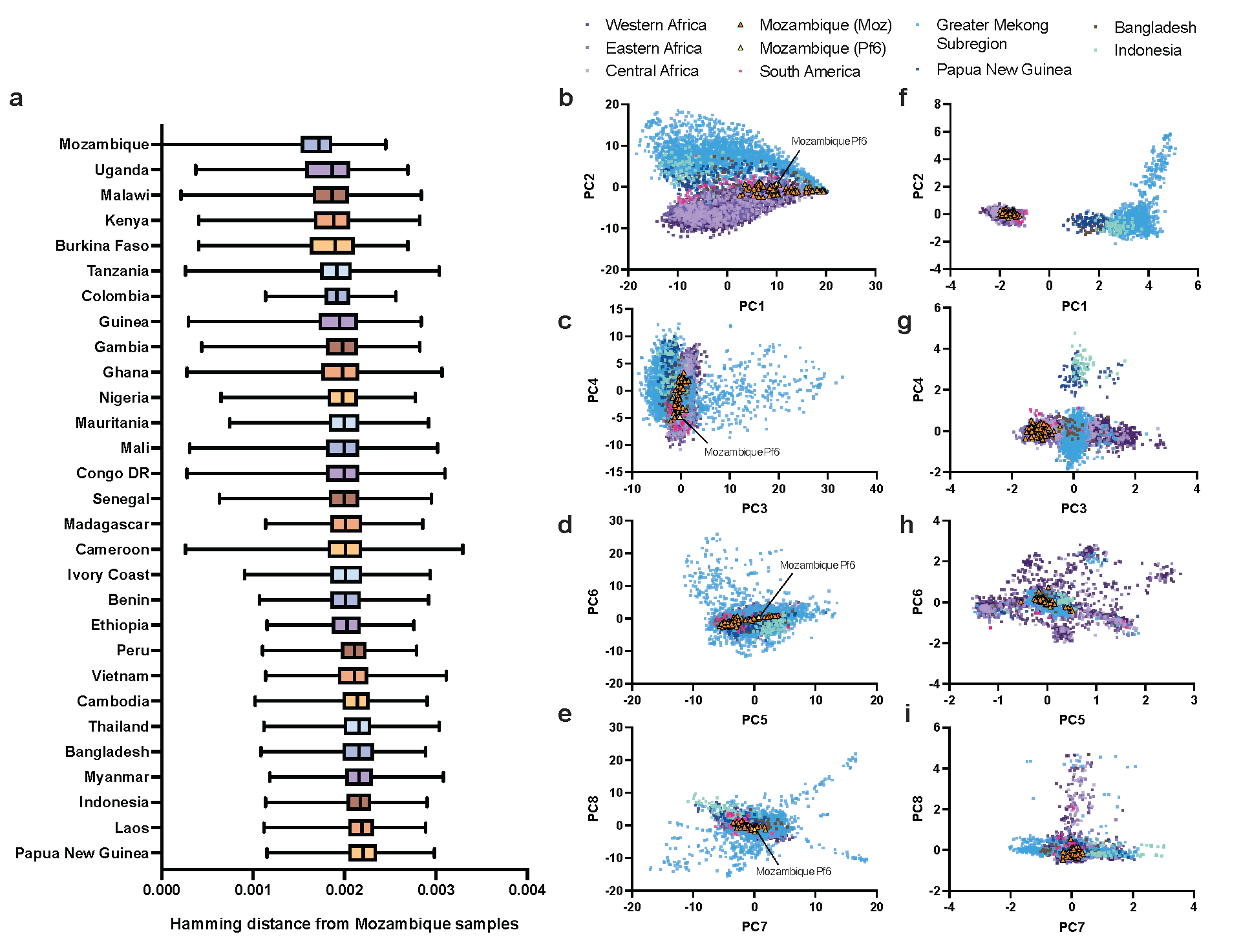

Supplement: Fig. S2 — Comparison to Pf6 samples using approximation of genetic distance and PCA. [file mbio.01768-23-s0002.tif]

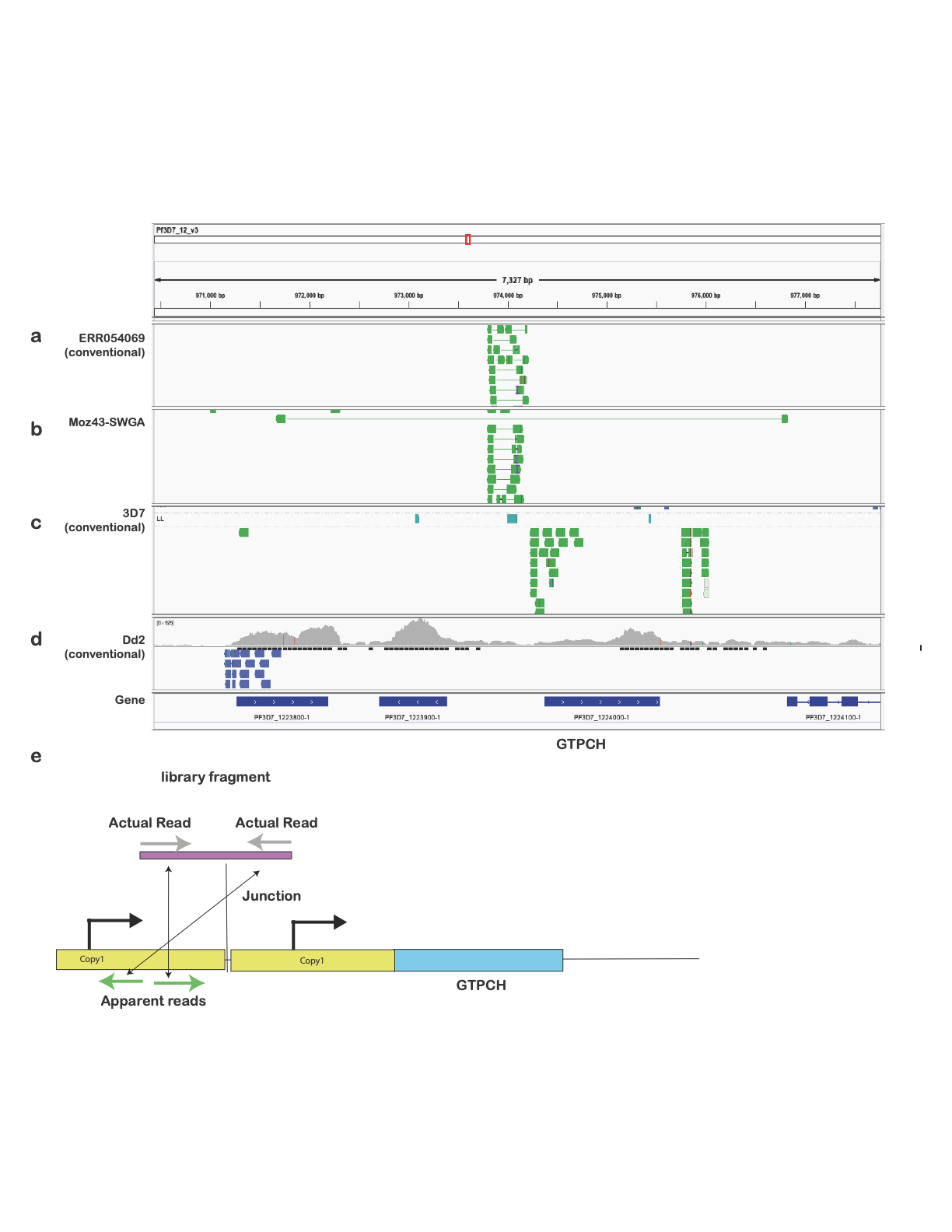

Supplement: Fig. S3 — Paired-end discordant read evidence of a 5kb tandem duplication event involving GTP cyclohydrolase on chromosome 12. [file mbio.01768-23-s0003.tif]

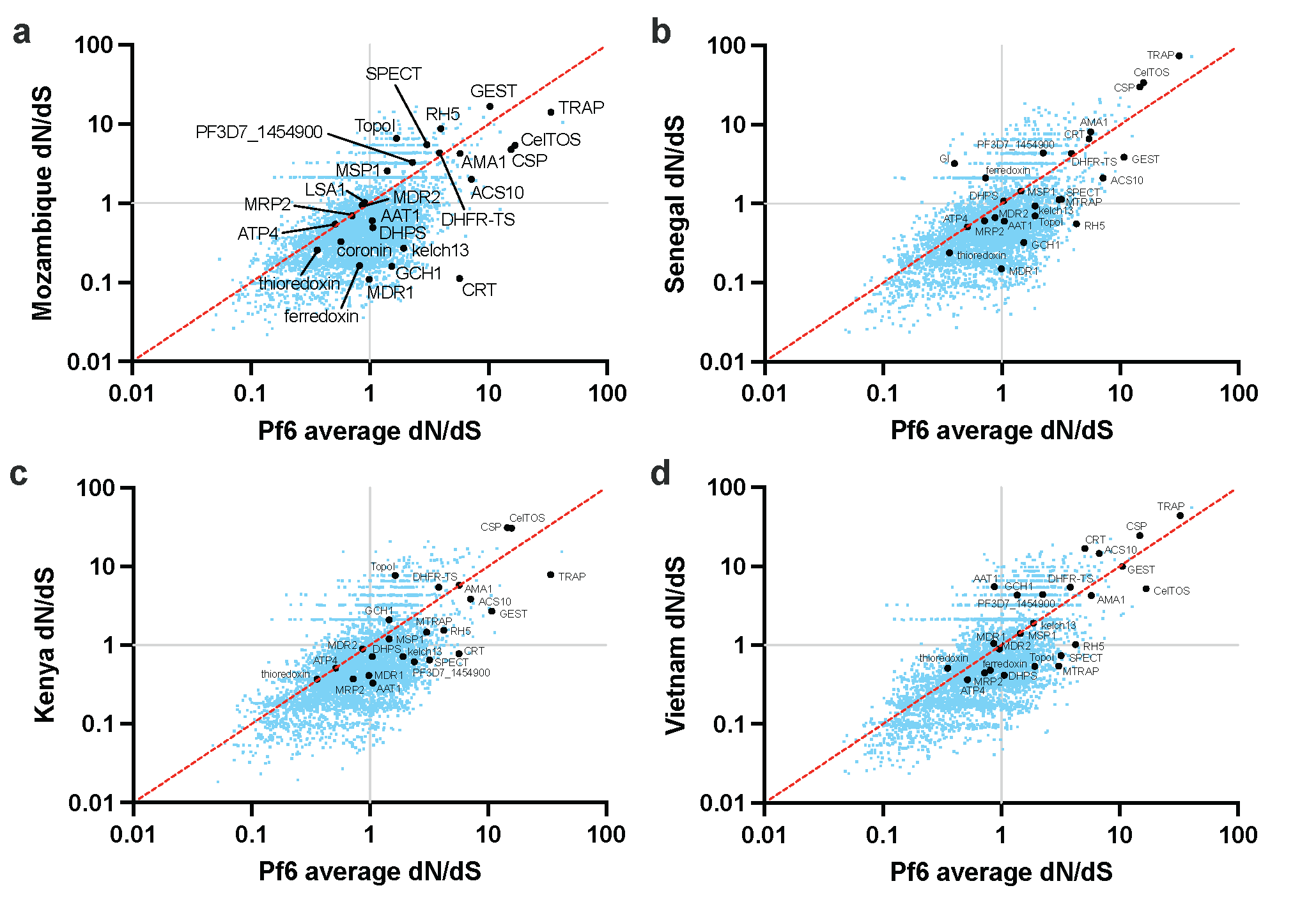

Supplement: Fig. S4 — dN/dS comparisons between specific countries (Mozambique, Senegal, Kenya, and Vietnam) and worldwide averages from Pf6. [file mbio.01768-23-s0004.tif]
