# Supplementary material for: Whole-genome surveillance identifies markers of Plasmodium falciparum drug resistance and novel genomic regions under selection in Mozambique
Source: mBio. 2023 Sep 26;14(5):e01768-23. doi: 10.1128/mbio.01768-23 (PMC10653802; doi:10.1128/mbio.01768-23)
Supplement: Supplemental material — Supplemental legends, Table S1, and Table S3. [file mbio.01768-23-s0005.docx]

**Supplemental Materials**

**Title:** Whole genome surveillance identifies markers of *Plasmodium falciparum* drug resistance and novel genomic regions under selection in Mozambique

**Contents:**

**Figure S1.** Mean coverage vs. Parasitemia.

**Figure S2**. Comparison to Pf6 samples using approximation of genetic distance and PCA.

**Figure S3**. Paired-end discordant read evidence of a 5kb tandem duplication event involving GTP cyclohydrolase on chromosome 12.

**Figure S4**. dN/dS comparisons between specific countries (Mozambique, Senegal, Kenya and Vietnam) and worldwide averages from Pf6.

**Table S1:** Coverage statistics before and after SWGA.

**Table S2.** Clonality calculations. Link to .xls file.

**Table S3:** Coverage across *pfcrt* and *pfmdr1*

**Fig. S1:** **Mean coverage vs. Parasitemia**. Relationship between average coverage (*P. falciparum* sequences) and parasitemia for 120 samples. Parasitemia was determined by microscopy and mean coverage calculations were performed in Samtools.

**Fig. S2:** **Comparison to Pf6 samples using approximation of genetic distance and PCA**. **(a)** Hamming distance of rounded alternate allele frequencies (threshold 0.5) for 61,630 sites with minimum coverage of 20 in all 4,352 samples with sufficient coverage from our Mozambique isolates and the Pf6 dataset (see Methods). **(b-e)** Plots for pairs of the top eight principal components from PCA performed on binary encoding of alternate allele presence in 459,725 core nuclear genome sites in 6,686 samples, including Mozambique isolates and the Pf6 dataset. A universal strict threshold of ≥0.9 alternate allele frequency and ≥20 alternate allele depth was used for the binary encoding. **(f-i)** Plots of the top eight principal components from PCA performed on the rounded allele frequencies for the 61,630 sites and 4,352 samples described in (a).

**Figure S3**. **Paired-end discordant read evidence of a 5kb tandem duplication event involving GTP cyclohydrolase on chromosome 12.** Evidence of duplication in the GTP cyclohydrolase (GTPCH) promoter region in **(a)** a conventionally sequenced clone from Africa(1) and **(b)** A Mozambique sample from this dataset amplified via SWGA, compared to **(c)** the 3D7 *P. falciparum* reference line with a tandem duplication and **(d)** the Dd2 *P. falciparum* reference line with an inverted duplication. Tracks are shown with groupings by read orientation in Integrative Genomics Viewer (IGV). Green read tracks are reversed right-left orientation (RL), the blue are RR. Not shown are proper (LR) and LL orientations (observed in Dd2 around position 976213). The data confirms that 3 genes are amplified in Dd2 and only GTPCH in 3D7(3) but that the repeat is an inverted duplication in Dd2 and tandem in 3D7 **(e)** Schematic depicting the detection of CNVs post WGS and SWGA as outward facing reads with insert sizes larger than expected. The amplification in the promoter region GTPCH in African samples has also been reported by others(2).

**Fig. S4:** **dN/dS comparisons between specific countries and worldwide averages from Pf6.** dN/dS (ratio of nonsynonymous (NS) to synonymous SNV counts normalized by expected numbers of NS and S sites under a neutral model; see Methods) of 4,468 genes containing at least one SNV for four countries—**(a)** Mozambique, *n*=94 samples included, 93 of which are from the present study; **(b)** Senegal, *n*=84; **(c)** Kenya, *n*=110; and **(d)** Vietnam, *n*=226—in comparison to dN/dS averaged over all other countries in the combined Mozambique and Pf6 dataset. Only Pf6 QC pass samples and Mozambique samples with coverage ≥5 were considered for dN/dS calculations.

**Table S1: Coverage statistics before and after SWGA.** A subset of samples was processed with and without SWGA prior to WGS to assess effect on coverage and sequencing quality without amplification. SWGA increased the average % callable genome (% covered by ≥5 reads) from less than 15% to over 75% allowing for accurate variant calling.

| Sample ID: | Total reads: | Aligned reads: | % Reads aligned | Mean coverage: | % Bases covered by ≥5 reads: |
| --- | --- | --- | --- | --- | --- |
| Moz-1 | 29495352 | 20877891 | 0.707837 | 65.18 | 94.5 |
| Moz-2 | 31985484 | 29832164 | 0.932678 | 93.76 | 96.4 |
| Moz-3 | 60231932 | 4919005 | 0.081668 | 9.91 | 45.5 |
| Moz-4 | 52175470 | 2573791 | 0.04933 | 4.28 | 21.2 |
| Moz-5 | 17881576 | 13673965 | 0.764696 | 42.4 | 91.3 |
| Moz-7 | 25832626 | 23861745 | 0.923706 | 70.15 | 95.3 |
| Moz-8 | 12750366 | 11287733 | 0.885287 | 37.15 | 87.7 |
| AVERAGE | **32907543.70** | **15289470.6** | **0.62** | **46.12** | **75.99** |
| Moz-1-UnAmp | 79658750 | 1945795 | 0.024427 | 1.99 | 11.9 |
| Moz-2-UnAmp | 44791280 | 2554928 | 0.057041 | 5.44 | 51.9 |
| Moz-3-UnAmp | 45485226 | 943089 | 0.020734 | 0.76 | 4.3 |
| Moz-4-UnAmp | 62941084 | 1360520 | 0.021616 | 1 | 5.7 |
| Moz-5-UnAmp | 39964942 | 720115 | 0.018019 | 0.63 | 3 |
| Moz-7-UnAmp | 26517654 | 1109974 | 0.041858 | 2.13 | 16.1 |
| Moz-8-UnAmp | 120316754 | 2459024 | 0.020438 | 1.88 | 10.1 |
| AVERAGE | **59953670** | **1584777.86** | **0.03** | **1.976** | **14.71** |

**Table S2:** **Clonality calculations**. Separate .xls file.

**Table S3: Coverage across *pfcrt* and *pfmdr1*.** Coverage statistics provided by samtools coverage command for chr7:403K-407K(pfcrt) and chr5:957500-962500(pfmdr1) across samples.

| C’some | Start pos | End pos | Num reads | Covered bases | Coverage | Mean depth | Mean base Q | Mean map Q |
| --- | --- | --- | --- | --- | --- | --- | --- | --- |
| chr5:957500-962500(*pfmdr1*) | | | | | | | | |
| Pf3D7_05_v3 | 957890 | 962149 | 149398 | 4260 | 100 | 3326.1 | 25.4 | 59.3 |
| chr7:403K-407K(*pfcrt*) | | | | | | | | |
| Pf3D7_07_v3 | 403222 | 406317 | 143429 | 3096 | 100 | 4127.28 | 25.1 | 57.7 |

1. Amambua-Ngwa A, Amenga-Etego L, Kamau E, Amato R, Ghansah A, Golassa L, Randrianarivelojosia M, Ishengoma D, Apinjoh T, Maiga-Ascofare O, Andagalu B, Yavo W, Bouyou-Akotet M, Kolapo O, Mane K, Worwui A, Jeffries D, Simpson V, D'Alessandro U, Kwiatkowski D, Djimde AA. 2019. Major subpopulations of Plasmodium falciparum in sub-Saharan Africa. Science 365:813-816.

2. Turkiewicz A, Manko E, Sutherland CJ, Diez Benavente E, Campino S, Clark TG. 2020. Genetic diversity of the Plasmodium falciparum GTP-cyclohydrolase 1, dihydrofolate reductase and dihydropteroate synthetase genes reveals new insights into sulfadoxine-pyrimethamine antimalarial drug resistance. PLoS Genet 16:e1009268.

3. Kidgell C, Volkman SK, Daily J, Borevitz JO, Plouffe D, Zhou Y, Johnson JR, Le Roch K, Sarr O, Ndir O, Mboup S, Batalov S, Wirth DF, Winzeler EA. 2006. A systematic map of genetic variation in Plasmodium falciparum. PLoS Pathog 2:e57.
